# MDiGest: A Python package for describing allostery from molecular dynamics simulations

**DOI:** 10.1063/5.0140453

**Published:** 2023-06-05

**Authors:** Federica Maschietto, Brandon Allen, Gregory W. Kyro, Victor S. Batista

**Affiliations:** Department of Chemistry, Yale University, 225 Prospect Street, New Haven, Connecticut 06520, USA

## Abstract

Many biological processes are regulated by allosteric mechanisms that communicate with distant sites in the protein responsible for functionality. The binding of a small molecule at an allosteric site typically induces conformational changes that propagate through the protein along allosteric pathways regulating enzymatic activity. Elucidating those communication pathways from allosteric sites to orthosteric sites is, therefore, essential to gain insights into biochemical processes. Targeting the allosteric pathways by mutagenesis can allow the engineering of proteins with desired functions. Furthermore, binding small molecule modulators along the allosteric pathways is a viable approach to target reactions using allosteric inhibitors/activators with temporal and spatial selectivity. Methods based on network theory can elucidate protein communication networks through the analysis of pairwise correlations observed in molecular dynamics (MD) simulations using molecular descriptors that serve as proxies for allosteric information. Typically, single atomic descriptors such as α-carbon displacements are used as proxies for allosteric information. Therefore, allosteric networks are based on correlations revealed by that descriptor. Here, we introduce a Python software package that provides a comprehensive toolkit for studying allostery from MD simulations of biochemical systems. *MDiGest* offers the ability to describe protein dynamics by combining different approaches, such as correlations of atomic displacements or dihedral angles, as well as a novel approach based on the correlation of Kabsch–Sander electrostatic couplings. *MDiGest* allows for comparisons of networks and community structures that capture physical information relevant to allostery. Multiple complementary tools for studying essential dynamics include principal component analysis, root mean square fluctuation, as well as secondary structure-based analyses.

## I. INTRODUCTION

Allostery is a regulatory process of biomolecules that propagates the effect of an event at an allosteric site and causes a change in conformation or activity at a distant site. Allosteric mechanisms are ubiquitous in proteins and protein-nucleic acid complexes, where cooperativity leads to conformational changes spanning multiple domains.^1^ Understanding allosteric regulation in biomolecules is challenging since the nature and scope of allosteric interactions are extremely varied. Structural and/or dynamic changes are transferred across the macromolecule through conformational changes often described by a complex interplay of local contacts and collective motions occurring over multiple timescales. Therefore, elucidating allosteric mechanisms often requires a combination of experimental and computational methodologies. Here, we introduce a Python software package that provides a comprehensive toolkit for studying allostery from molecular dynamics (MD) simulations of biochemical systems.

Many allostery-related studies are based on conformational ensembles obtained from molecular dynamics (MD) simulations combined with experimental data from nuclear magnetic resonance (NMR) spectroscopy enabling atomic-level characterizations of protein motions. Correlated motions are typically inferred from the statistical analysis of thermal nuclear fluctuations and from the NMR spectroscopic analysis, assisted by mutagenesis and kinetic assays.

The statistical analysis of correlations often includes the use of mutual information (MI)-based correlation metrics^2,3^ as well as variance-covariance-based approaches,^4,5^ providing access to both local perturbations and collective motions that can reveal a detailed picture of allosteric communications in proteins. Such methodologies have been shown to provide valuable insights into experimental data for various allosteric enzymes.^6,7^ Efficient and flexible computational tools are highly desired to gain fundamental insights about the residues involved in allosteric regulation and their influence on dynamics and protein function, prompting many recent developments devoted to such a purpose.^8–18^ Such tools are essential for the analysis of long-time simulations of large systems involving the motion of large domains, making it difficult to identify suitable descriptors to describe the underlying allosteric pathways.^19^

One of the most important considerations is the frame of reference for extracting the motion. If cartesian coordinates are chosen, the first difficulty is choosing the alignment frame, as it has been shown that a poor choice of reference frame can lead to artifactual conclusions.^21^ The choice of internal coordinates is natural for proteins because they are the rotational and translational invariants.^22^ Relative distances, dihedrals, and structural alphabets are examples of popular metrics to investigate allosteric control in biological macromolecules,^23–26^ although they are not exempt from limitations. Dihedrals, for instance, can characterize protein conformational changes through dihedral PCA (dPCA),^23,27^ but they require careful selection of the dihedral space and can be difficult to analyze in proteins with several secondary structure elements. The difficulties are related to the so-called “loop closure problem,” since adjacent backbone dihedral angles are necessarily correlated in proteins with relatively rigid secondary structures.^28^ As a result, nonlinear correlations may render an inefficient representation of the dynamics of dihedral angles, particularly when the large-amplitude functional motions of proteins are considered. In these cases, other choices of internal coordinates, such as contacts or pairwise distances,^26,29^ might be advantageous. The former, however, requires careful selection of optimal parameters. For example, a contact-persistency cutoff defines contact interactions as determined by the percentage of configurations where two atoms are found closer than a distance cutoff.

The challenging aspect of such approaches remains the choice of the parameters so that the results are stable and have a valuable physical meaning. Recently, we introduced a new metric based on electrostatic interactions,^30^ which has the advantage of being independent of the choice of alignment frame while still being defined by pairwise distances that determine the strength of coupling interactions. As such, the electrostatic metric is complementary to other descriptors, especially in the context of multidomain complexes where the predicted couplings are strongly dependent on the choice of reference structure or contact map parameters.

Here, we introduce the Python package *MDiGest* to analyze MD simulations and identify key amino acid residues involved in allosteric pathways. *MDiGest* is built upon MDAnalysis,^31^ which is a widely used trajectory handler package, allowing users to analyze MD results in popular formats such as DCD, TRR, or CRD with no pre-processing. We focus on extending the applicability of the network analysis methodology through the *MDiGest* interface and its implementation, analyzing the reproducibility of results using replicas of targeted systems, and ultimately improving our ability to interpret results from the vast amount of raw data gathered from MD simulations.

*MDiGest* offers capabilities beyond those of previously distributed packages where correlations are calculated according to specific “features,” with the atom selection often being restricted to backbone coordinates or torsion angles. *MDiGest* offers multiple options, including not only backbone coordinates and torsion angles but also the analysis of correlations based on electrostatic couplings. Moreover, *MDiGest* includes several correlation measures so that different metrics can be analyzed comparatively through their respective allosteric networks.

*MDiGest* is offered in terms of Jupyter tutorial notebooks and Python modules, making it easy and practical to use. Additionally, *MDiGest* prepares input scripts for PyMOL,^20^ allowing for the practical rendering of biomolecular images.

**FIG. 1.**
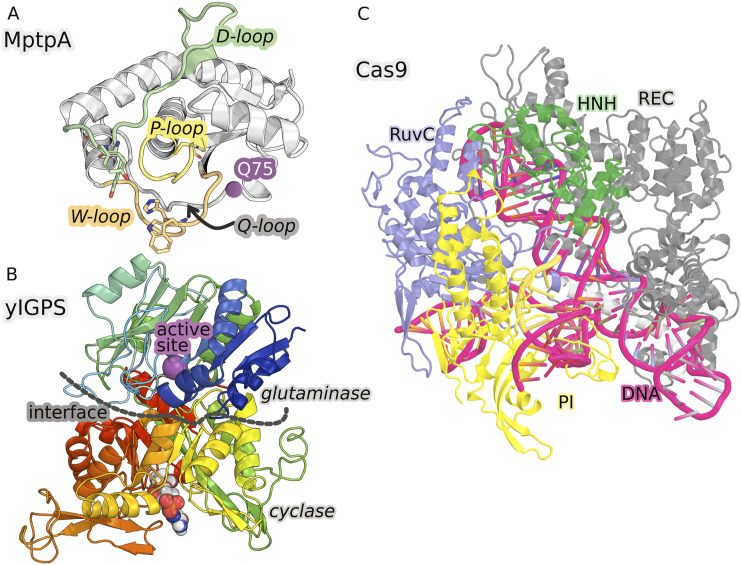
Biomolecular model systems used to demonstrate the capabilities of the *MDiGest* software package as applied to modeling allostery. (a) Structure of the MptpA enzyme with three catalytic loops, including the P-loop (yellow cartoon) with the catalytic cysteine 11 (C11), surrounded by the acid loop (green), and the W-loop (orange). The distance between W48 and Y128, Y129 (in sticks) distinguishes the open and closed states of the enzyme. The Q75 residue is shown in purple. (b) Structure of yeast IGPS, including the glutaminase and cyclase enzymatic domains separated by a dashed line. (c) Structure of the Cas9 complex, with the protein and nucleic acid structures shown in new cartoon representations, while ligands are shown with sphere representations. Images were rendered using PyMOL.^20^

The capabilities of *MDiGest* are demonstrated as applied to the analysis of allostery in three representative biological systems (Fig. 1), including the protein tyrosine phosphatase (PTP) enzyme (MptpA), the imidazole glycerol phosphate synthase (IGPS) enzyme, and the Clustered Regularly Interspaced Short Palindromic Repeats associated protein 9 (CRISPR-Cas9) enzyme, a multi-subunit dual RNA-guided DNA endonuclease enzyme.

**FIG. 2.**
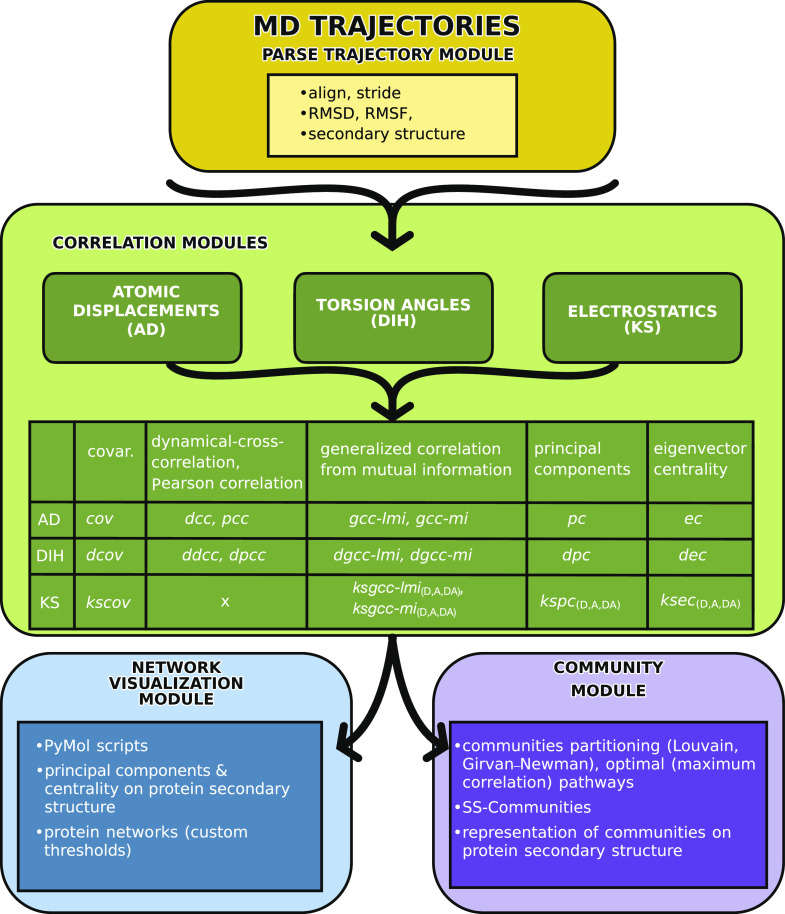
The modular structure of the *MDiGest* software. The trajectory is processed through the *parsetrajectory* module. Three correlation modules can be used to compute several correlation metrics and related metrics from the atomic displacements, torsion angles (denoted with the prefix *d*), and electrostatic energy couplings (denoted with the prefix *ks*), respectively, as reported in the diagram. The acronyms are described in Sec. II. The resulting correlation matrices can be used as input for the visualization and community modules. The visualization module produces PyMol^20^ scripts for visualizing the projected properties onto the protein structure. In addition, the correlation matrices can be passed to the community module to partition the protein graphs into community structures.

### A. Protein-tyrosine phosphatase enzyme (MptpA)

MptpA is ideally suited for illustrating the analysis of allostery provided by *MDiGest*. Protein-tyrosine phosphatases (PTPs) and protein-tyrosine kinases co-regulate cellular processes. In pathogenic bacteria, PTPs are frequently involved as key virulence factors for human diseases.^32^ For example, the *Mycobacterium tuberculosis* organism responsible for tuberculosis secretes the low molecular weight enzyme protein-tyrosine phosphatase MptpA, which is required for survival upon infection of host macrophages. Its phosphate-binding loop (P-loop) CX5R and the loop containing a critical aspartic acid residue (D-loop) required for the catalytic activity are conserved, although there is otherwise no sequence similarity between this enzyme and other classes of PTPs.

In most high molecular weight PTPs, as well as in MptpA, 30 ligand binding to the P-loop triggers a large conformational reorientation of the D-loop, changing from an “open” to a “closed” conformation upon displacement by about 10 Å. The section *Test Case 1*, *SI*, of the *mdigest-tutorial-notebook.ipynb* includes the analysis of the allosteric regulation of such conformational change based on three independent MD trajectories of WT MptpA and the Q75L mutant. The nature of the allosteric mechanism provides an understanding of the enhanced catalytic activity in the Q75L mutant at the molecular level.^30^

The closed form of MptpA is stabilized by strong electrostatic interactions between the W-loop and the adjacent Q-loop. The mutant Q75L has been found to enhance its catalytic activity, revealing Q75 to be an allosteric modulator.^30^ Recently,^30^ we have shown that MD simulations support the experimental evidence of increased catalytic activity for the Q75L mutant.

### B. Imidazole glycerol phosphatase synthase (IGPS) enzyme

IGPS is another benchmark model that serves to demonstrate the capabilities of *MDiGest*. IGPS is a key metabolic enzyme of the amidotransferase family that links amino acid and nucleotide biosynthesis in bacteria, plants, and fungi but is absent in mammals. Because of its metabolic role, IGPS is also a potential target for herbicides and antifungal agents.^33^ The IGPS from *S. cerevisiae* (yIGPS) consists of a single 124 kDa subunit (His7) with two enzymatic subunits, including the glutaminase (residues 1–235) and cyclase (residues 239–552) domains [Fig. 1(b)].^34^ yIGPS catalyzes glutamine (Gln) hydrolysis at the active site of the glutaminase domain, where there is a conserved catalytic triad (C83, H193, and E195) that produces ammonia (NH_3_) and glutamate. The generated NH_3_ travels from the glutaminase active site to the cyclase effector site more than 25 Å away and reacts to break down the N′-[(5′-phosphoribulosyl)formimino]-5-aminoimidazole-4-carboxamide ribonucleotide (PRFAR) effector into two products [imidazole glycerol phosphate (IGP) and 5-aminoimidazole-4-carboxamide ribonucleoside (AICAR)], essential precursors for the histidine and purine biosynthetic pathways, respectively.^35–37^ The glutaminase half-reaction of yIGPS exhibits V-type allosteric modulation, with the Gln binding affinity being minimally altered by the presence of PRFAR, while a ∼5000-fold increase in reaction rate is observed for the PRFAR-bound complex over the basal glutaminase activity of yIGPS.^36,37^ Here, we show the capabilities of *MDiGest* to perform a community network analysis revealing how the binding of the effector PRFAR alters the early allosteric dynamics.

### C. CRISPR-Cas9 complex

Here, we focus on the prokaryotic Clustered Regularly-Interspaced Short Palindromic Repeats associated protein 9 (CRISPR-Cas9) complex (with 1362 residues) as a model system that serves to test the capabilities of *MDiGest* as applied to a large and truly complex allosteric enzyme. The analysis of allostery is focused on understanding the precise mode of communication from the alpha-helical recognition (REC) domain to the HNH nuclease domain, an important aspect that is critical for specificity. The CRISPR-Cas9 system has been adapted as a powerful and versatile DNA-targeting platform that enables the engineering of genomes with unprecedented functionality.^38,39^ The most widely used Cas9 from *Streptococcus pyogenes* (Cas9 hereafter) is a dual RNA-guided, multidomain endonuclease [Fig. 1(c)].^40,41^ Cas9 is complexed with a chimeric single guide RNA [Fig. 1(c)] to perform its double-stranded DNA targeting and cleavage function. It can be programmed with the corresponding 20-nucleotide guide sequence at the 5′ end of sgRNA to target any genomic site flanked by a short protospacer adjacent motif (PAM).^42–44^ The apo-Cas9 structure adopts a bi-lobed architecture and comprises an alpha-helical recognition (REC) lobe and a nuclease (NUC) lobe connected by an arginine-rich bridge helix (BH) [Fig. 1(c)]. The REC lobe is further divided into three domains (REC1, REC2, and REC3), and the NUC lobe consists of a PAM-interacting (PI) domain and two Mg^2+^-dependent nuclease domains (HNH and RuvC). HNH and RuvC function as molecular scissors, cutting the target strand complementary to the sgRNA 5′ end and the nontarget strand of a dsDNA target, respectively. Therefore, Cas9 is a complex allosteric enzyme that undergoes a sequence of precise conformational rearrangements necessary for DNA target recognition and cleavage.^44–46^ These processes involve multiple layers of allosteric regulation to ensure targeting precision and functional activity.^39,44,46,47^

## II. THEORY

Graph theory is the study of mathematical structures called graphs consisting of nodes and edges used to model pairwise relations between objects. The origins of graph theory date back to the eighteenth century, when Euler solved the dilemma of the Seven Bridges of Königsberg. Since then, network models have been widely applied to questions in a variety of fields, including the physical and biological sciences. Here, we focus on graphs of proteins where the nodes correspond to amino acid residues and the edges are defined according to the strength of pairwise correlations established among interacting residues.^48^ Protein networks have been traditionally generated using a variety of models, including protein correlation networks (PCNs),^2^ protein structure networks,^49^ protein contact networks,^50^ residue interaction graphs,^9,51^ and residue networks.^52^ In PCNs, the network is constructed from the correlation of time-dependent variables from MD simulations. The entries of the correlation matrix become the edges of undirected networks, with the nodes corresponding to the elements for which the correlation is computed.

### A. Node-level descriptors

We focus on allosteric communication in proteins, which is analyzed using correlation networks obtained from MD simulations of atomic displacements, torsion angles, and electrostatic energies (Fig. 2). To construct these networks, F snapshots of protein configurations (frames) are sampled at equidistant time steps, each including N nodes representative of the protein structure. Section II A outlines the structure of the modules that perform the correlation analysis for different features in MDiGest and details the size of the matrices/tensors involved in the calculation of each descriptor. When built from electrostatic energy couplings or torsion angles, protein networks preserve 1:1 node-to-residue mapping. Instead, the network constructed from atomic displacements may, in principle, exceed the number of residues in the system, depending on the user’s selection. However, user selections describing each residue as a single tridimensional vector are more easily interpretable and should be the preferred choice. For the sake of clarity, in the following, we assume that the number of nodes in the network equates to the number of residues in the system.

#### 1. Atomic displacements

Cartesian atomic displacements of residue *i* are computed for each frame of the MD simulation as follows:

$$r_{i}=q_{i}-\frac{1}{F}\sum_{j}^{F}q_{j},$$ (1)

resulting in a matrix of size *F* × 3*N* called the *atomic displacement matrix 1* (*adm1*), with *N* the number of atoms included in the correlation analysis and ***q***_***i***_ = {*x*_*i*_, *y*_*i*_, *z*_*i*_,} the coordinates of each node *i* (e.g., alpha carbon of each amino acid residue). The displacement magnitudes are computed as follows:

$$\|r_{i}\|=\sqrt{{\left(x_{i}-\bar{x}\right)}^{2}+{\left(y_{i}-\bar{y}\right)}^{2}+{\left(z_{i}-\bar{z}\right)}^{2}},$$ (2)

resulting in a matrix of size *F* × *N*, called *atomic displacement matrix 2* (*adm2*), where each element corresponds to the atomic displacement of a given amino acid residue for each frame of the MD simulation.

#### 2. Torsion angles

Four torsional coordinates, including sine and cosine backbone torsions and dihedrals, are recorded for each amino acid residue along the MD simulation in the matrix *torsion angle matrix 1* (*tam1*) of size *F* × 4*N*. Each matrix entry corresponds to the sine/cosine transformed/backbone dihedral for a given residue and snapshot of the MD simulation,

$$d_{i}=\left\{\sin\left(\varphi_{i}\right),\cos\left(\psi_{i}\right),\sin\left(\varphi_{i}\right),\cos\left(\psi_{i}\right)\right\}.$$ (3)

The magnitude of the dihedral displacement is computed as follows:

$$\|d_{i}\|=\sqrt{\sum_{k}^{4}{\left(d_{i,k}-\bar{d_{i}}\right)}^{2}},$$ (4)

resulting in a matrix of size *F* × *N* called *torsion angle matrix 2* (*tam2*).

#### 3. Electrostatic energies

The analysis of correlations of electrostatic energies is based on one descriptor per amino acid residue, corresponding to either the hydrogen bond donor energy, hydrogen bond acceptor energy, or the sum of the donor and acceptor energies according to the Kabsch–Sander formalism.^53^ We focus on electrostatic interactions between the CO and NH backbone groups for each frame of the MD simulation, as follows:

$$E=0.42e\times 0.20e\times 33.2\frac{kcal}{mol\times nm}\times \left(\frac{1}{r_{ON}}+\frac{1}{r_{CH}}-\frac{1}{r_{OH}}-\frac{1}{r_{CN}}\right),$$ (5)

resulting in a 3-mode tensor of size *F* × *N* × *N*, where rows correspond to hydrogen bond acceptor (CO) groups and columns correspond to hydrogen bond donor (NH) groups. From this matrix, we can sum across rows to obtain the hydrogen bond donor energy of each residue, resulting in the *Kabsch*–*Sander donor matrix* (*ksdm*) of size *F* × *N*, where each element is the hydrogen bond donor energy for a given residue and frame of the MD simulation. Analogously, summing across columns yields the *Kabsch*–*Sander acceptor matrix* (*ksam*) corresponding with the acceptor energy for a given residue and frame of the MD simulation. Additionally, we can sum the donor and acceptor energies for each residue, resulting in the *Kabsch*–*Sander donor-acceptor matrix* (*ksdam*) of size *F*′ × *N*, where each element is the sum of hydrogen bond donor and acceptor energies for each residue and frame of the MD simulation.

### B. Pairwise correlations

*MDiGest* allows for calculations of residue–residue couplings using four possible measures of correlation. Dynamic cross-correlation, Pearson correlation, generalized correlation based on the linearized mutual information, and generalized correlation obtained from the mutual information are computed from each of the three descriptors of amino acid residues, including atomic displacements, torsion angles, and electrostatic energies.

#### 1. Dynamic cross-correlation and Pearson correlation

The dynamic cross-correlations (*dcc*) between residues *i* and *j* are computed for the *F* frames of an MD simulation according to the following equation:

$$dcc_{i,j}=\frac{{\left\langle \Delta r_{i}\left(t\right)\Delta r_{j}\left(t\right)\right\rangle }_{t}}{\sqrt{{\left\langle \|\Delta r_{i}\left(t\right){\|}^{2}\right\rangle }_{t}}\sqrt{{\left\langle \|\Delta r_{j}\left(t\right){\|}^{2}\right\rangle }_{t}}}.$$ (6)

The *dcc* matrices are of size *F* × c*N*, where c is either 3 (for atomic coordinates), 4 (for torsion angles), or 1 (for electrostatic energies) as provided by matrices *adm1*, *tam1*, and *ksdam*, resulting in matrices of sizes *3N* × 3*N*, *4N* × 4*N*, and *N* × *N*, respectively. The matrices *adm1* and *tam1* are then averaged to obtain an *N* ′ *N* pairwise *dcc* coefficient.

We compute Pearson correlation coefficients (*pcc*) from *adm2*, *tam2*, and *ksdam* as follows:

$$pcc_{x,y}=\frac{\overset{F}{\underset{i}{\sum }}\left(x_{i}-\bar{x}\right)\left(y_{i}-\bar{y}\right)}{\sqrt{\overset{F}{\underset{i}{\sum }}{\left(x_{i}-\bar{x}\right)}^{2}\overset{F}{\underset{i}{\sum }}{\left(y_{i}-\bar{y}\right)}^{2}}},$$ (7)

where each element is the respective pairwise correlation coefficient.

#### 2. Generalized correlation

Generalized correlation coefficients (*gcc*) are computed from either the linearized mutual information or the mutual information, as follows:

$$gcc_{i,j}=\sqrt{1-\exp\left(-\frac{2}{3}I_{i,j}\right)}.$$ (8)

Using a Gaussian estimator, one can approximate the (linearized) mutual information^2^ between two variables as follows:

$$I_{i,j}=\frac{1}{2}\left[\log\left(\det\left(cov_{i}\right)\right)+\log\left(\det\left(cov_{j}\right)\right)-\log\left(\det\left(cov_{i,j}\right)\right)\right],$$ (9)

where the covariances are computed from the matrices *adm1*, *tdm1* or *ksdm*, *ksam*, *ksdam* for each pair of descriptors *i*, *j*, as follows:

$$cov_{x,y}=\frac{\overset{F}{\underset{i}{\sum }}\left(x_{i}-\bar{x}\right)\left(y_{i}-\bar{y}\right)}{F}.$$ (10)

The resulting matrix accounts for only linear correlations. Therefore, the corresponding correlation matrix is referred to as *gcc-lmi*. Additionally, we can compute *gcc-mi* coefficients from the mutual information,

$$I_{i,j}=H_{i}+H_{j}-H_{i,j},$$ (11)

that also accounts for nonlinear correlations. Following the derivation by Kraskov *et al.*,^54^ we approximate the mutual information as follows:

$$I_{i,j}\approx \psi_{l}-\frac{1}{k}-\left\langle \psi_{n_{i}},+,\psi_{n_{j}}\right\rangle +\psi_{F},$$ (12)

where *k* is a parameter defining the number of nearest neighbors, *F* is the total number of frames in the MD simulation, and are the numbers of frames in which the positions of nodes *i* and *j* are within a specified distance cutoff. The digamma function introduced by Eq. (12) is defined as follows:

$$\psi_{l}=\Gamma_{l}^{-1}\frac{d\Gamma_{l}}{dl},$$ (13)

where *l* = *k*, *F*, *n*_*i*_, *n*_*j*_. Furthermore, is the ensemble average of the sum of digamma functions applied to *n*_*i*_, *n*_*j*_, where *n*_*i*_, *n*_*j*_ are varied for each calculation of $\left\langle \psi_{n_{i}},+,\psi_{n_{j}}\right\rangle$ according to a distance cutoff, including k-nearest neighbors for each node *x* and *y* in each frame along the MD simulation.

In the implementation of the method, *MDiGest* first initializes an *N* × *N* array with each entry corresponding to k and F. To solve for this, we first define x to be an *F* × *f* matrix, where *f* is 3 for atomic displacements, 4 for torsion angles, and 1 for electrostatic energies for each node *i*. We define y analogously for node j. The concatenated [x,y] array of size 2*F* × *f* is fed into a nested KDTree object, utilizing the Chebyshev distance metric, i.e., the maximum absolute distance in one dimension of two *n*-dimensional points. The KDTree provides the distances to the *k*-nearest neighbors for each point. An additional KDTree object for each node (*i* and *j*, individually) is used to compute the digamma for the points in each of these two trees at all distance cutoffs defined by the outer KDTree. Once the average is computed, the mutual information between nodes *i* and *j* is readily obtained.

### C. Eigenvector centrality

The eigenvector centrality (*ec*) measures the relative importance of nodes in establishing correlations in the network.^55–57^ From each set of pairwise correlation coefficients, we can construct a corresponding adjacency matrix and obtain the eigenvector centralities of the nodes in the network as established by that specific correlation coefficient.

*MDiGest* allows for the construction of adjacency matrices, A, of size *N* × *N*, for each set of correlation coefficients (*dcc*, *pcc*, *gcc-lmi*, and *gcc-mi*) based on one of three atomic descriptors (atomic displacements, torsion angles, and electrostatic energies). Each entry of A is a pairwise correlation coefficient representing an edge between two nodes in the network. From the adjacency matrix, the eigenvector centrality is computed as follows:

Ac=λc. (14)

According to the Perron–Frobenius theorem, the entries of the eigenvector corresponding to the largest eigenvalue can be defined to be all positive real numbers, defining the centrality values for each node (residue) in the network (protein). Each entry of the eigenvector **c** with the maximum eigenvalue thus corresponds to the eigenvector centrality of the given node. The eigenvector centrality values, therefore, quantify the importance of each node in the eigenvector of maximum eigenvalue, measuring how much each node of the protein network contributes to the correlation in the network.

When the adjacency matrix to be diagonalized corresponds to the covariance matrix (*cov*) of atomic displacement atoms, the resulting set of transformed coordinates is usually referred to as the principal components (*pc*) of motion. The eigenvectors, or *pc*s, describe the motions of the system, and the corresponding eigenvalues report on the relative contribution of each motion to the global dynamics.

### D. Networks obtained with MdiGest

*MDiGest* allows the building of a variety of protein correlation networks for a comprehensive analysis of allostery in the system. As described in Sec. II A, correlations can be quantified by the covariance of atomic displacements, dynamic cross-correlation, Pearson correlation, the generalized correlation from linearized mutual information, and the generalized correlation from mutual information using various node-level descriptors, including atomic displacements, torsion angles, and electrostatic energies. As recently shown,^30^ the electrostatic eigenvector centrality (EEC) measure yields improved correspondence with the experimental analysis of allostery based on NMR data. The information provided by EEC generally resembles that obtained from correlations of backbone dihedrals, confirming that correlated electrostatic couplings account for both localized motions and overall conformational changes.^22^ Furthermore, the decomposition between donor and acceptor energies provides an additional layer for an in-depth interpretation of the underlying dynamics.

## III. MDiGest MODULES AND FEATURES

*MDiGest* is a Python 3 package that can be executed on Linux, Mac OS, and Windows operating systems. The installation may be accomplished directly using Conda or through pip, as described in https://github.com/fmaschietto/mdigest#readme. It is efficiently built using high-end and actively-maintained Python packages such as NumPy,^58^ SciPy,^59^ scikit-learn,^60^ NetworkX,^61^ *dynetan*,^18^ and MDAnalysis,^31^ The trajectory parsing in *MDiGest* is handled by MDAnalysis,^31^ which is among the most popular packages for such purposes, providing a reliable platform for a broad audience, including both the computational and experimental structural biology communities.

*MDiGest* is an object-oriented package consisting of different modules, each performing a specific function related to the processing/analysis of MD trajectories and the characterization of allosteric behavior. MD trajectory parsing is handled by the *mdigest.core.parsetrajectory* module, which allows customized parsing of trajectories, including slicing and frame selection across one or more replicates for a given system. Additionally, basic analyses are allowed, such as root-mean-square deviation (RMSD), root-mean-square fluctuation (RMSF), and calculation of secondary structure elements using the dictionary of protein secondary structure provided by MDTraj.^62^

Upon digestion by *mdigest.core.parsetrajectory*, the MD trajectories are stored in an object that allows easy access to MDAnalysis attributes as well as custom properties related to the replicates and slicing parameters. The modular structure (Fig. 2) allows for a progressive inheritance of relevant parent object instances that are used for subsequent analyses.

### A. Correlation modules and associated networks

*Atomic displacements*. The network constructed from pairwise correlations of atomic displacements enables the identification of the “hubs” in the protein network that play a major role in the dynamics of the system of interest. Different correlation matrices are used, including simple covariance, direct correlation coefficients, and linear (*lmi*)/non-linear (*mi*)-based correlations, each having different properties and, therefore, providing useful insights to individuate nodes (residues/atoms) that play a crucial role in the allosteric dynamics of the system. The *mdigest.core.correlation* module accounts for those procedures.

*Internal coordinates (i) Dihedral-based correlation network.* A complementary approach to atomic displacements utilizes dihedral angles to build the correlation network. Dihedral angles are advantageous in some instances because internal coordinates naturally provide a correct separation of internal and overall motions, which is crucial for the construction and interpretation of the pseudo-free energy landscape^22^ of a biomolecule undergoing large structural rearrangements. The *mdigest.core.dcorrelation* module allows one to generate a protein correlation network from dihedral fluctuations. To account for the circular statistics of angular variables, we transform the space of dihedral angles {*φ*_*n*_} to the metric coordinate space {*x*_*n*_ = cos *φ*_*n*_, *y*_*n*_ = sin *φ*_*n*_}. As such, each residue is described by four coordinates corresponding to the sine and cosine projections of the *φ* and backbone angles.

*Internal coordinates (ii) Electrostatic-based correlation network.* Pairwise electrostatic energies allow for decoupling the contributions of backbone donor (amide) and backbone acceptor (carbonyl) groups, providing valuable insights for the interpretation of dynamics. Electrostatic networks are computed through the *mdigest.core.kscorrelation* module. Each correlation module allows for the computation of the associated centrality metric.

A collection of pre-compiled plots in *mdigest.core.plots* can be used to generate heatmaps of the different PCNs, including plots corresponding to the difference network relative to two distinct states of a given system (i.e., ligand-bound vs ligand-unbound, wild-type vs mutant, etc.).

The *mdigest.core.savedata* module can be used to save the attributes of each class as binary files. The instances from each correlation analysis can be loaded back into a common object, which facilitates handling and post-processing analysis. Relevant examples are provided in the sections *Test Case 1* of the notebook *mdigest-tutorial-notebook.ipynb* (available at https://github.com/fmaschietto/mdigest/tree/master/notebooks), which shows the basic functionalities of the correlation related modules.

### B. Dimensionality reduction module

Eigenvector centrality belongs to the broader category of dimensionality reduction methodologies to extract “essential” information from MD trajectories. Computing centrality values based on eigenvector decompositions of similarity matrices reduces the dimensionality. When the decomposition involves the covariance matrix of a time series, the methodology is usually referred to as PCA. However, PCA differs from eigenvector centrality in that it is bound to linear correlations as a similarity metric. Given a set of mean-centered coordinates observed along an MD simulation, PCA sorts the trajectory into 3*N* directions of descending variance, with *N* being the number of atoms. These directions are called the principal components (*pc*s). The dimensions to be analyzed are reduced by only looking at a few projections of the first principal components. When the simulations are sufficiently long, the principal components with a multimodal probability distribution often correspond to distinct metastable conformational states, whereas unimodal probability distributions report on fluctuations rather than conformational transitions.^22,63^

Different correlation matrices and their corresponding protein networks and communities can be compared for a single replica as well as across multiple replicas.

The *mdigest.core.dimreduction* module can be used to perform such an analysis. Upon providing an MD trajectory (or, more generally, a time series of any variable), the linear transformation can be carried out using different methods, such as those provided through sklearn.decomposition.PCA^60^ and pyemma.coordinates.PCA.^64^ The resulting principal components are sorted in order of descending variance. Since the first few components usually capture the essential molecular motions of the MD simulation, it can be useful to project the original (centered) data onto the eigenvectors, allowing visualization of the essential motions along a given MD trajectory. The procedure is detailed in the associated notebook section (*Test Case 4 in mdigest-tutorial-notebook.ipynb*).

### C. Network visualization module

*MDiGest* includes a composite module (*mdigest.core.networkcanvas*) for generating visual representations of the resulting networks. Visualization is essential for studies of allostery with network models and for the interpretation of the underlying allosteric mechanisms.

Within the *ProcCorr* class, the user can load different correlation matrices and assign custom filters such that only entries falling within the desired threshold are selected. The generated subset of edges can be visualized on the protein secondary structure using a PyMOL^20^ script that is automatically generated by the class. More customized analyses and comparisons of different networks are provided by the standalone *draw_networks_pymol.py* module. Examples of how to load different correlation networks and represent them on the protein are provided in the section Test Case 3 of *mdigest-tutorial-notebook.ipynb.*

### D. Community partitioning module

A dynamic network has a community structure if the nodes of the network can be grouped into clusters that are internally well-connected. This topological analysis of PCNs can be used to partition the graph into communities and identify the significance of groups of residues with respect to the overall allosteric mechanism.

Community detection starts with a distance network, which can be constructed from a PCN using the equation d = −log(C), with C being the elements of the correlation matrix that constitute the edges of the network. *MDiGest* allows computation of the community structure of a PCN through the fast-performing Louvain heuristic scheme^65^ and the less efficient but more traditional Girvan–Newman (GN)^66^ algorithm (both in *the mdigest.core.networkcommunities* module). The Louvain heuristic outperforms the GN method when accounting for speed and accuracy,^18,67^ so it is the preferred choice. The quality of the partitioning is measured by modularity, where networks with high modularity have dense connections between the nodes within communities but sparse connections between nodes in different communities. The convergence can be analyzed across multiple runs or even across different correlation schemes. As described for the correlation analysis, the resulting community structures can be saved to file for later analysis. Relevant functionalities implemented in the community module are showcased in the section Test Case 4 of *mdigest-tutorial-notebook.ipynb*.

## IV. CASE STUDIES

The three systems introduced in Sec. I, including different levels of size and complexity, are discussed to demonstrate the capabilities of the *MDiGest* software. The section *Test Case 1* of the notebook introduces MptpA as a *tutorial system* for showcasing the calculation of correlations and relative networks. The small size of this enzyme and the experimental evidence that a specific mutant in a catalytically relevant loop perturbs its enzymatic activity render MptpA a great example for those who want to learn how to use network analysis tools to study allostery from MD data. Additionally, we apply a comprehensive repertoire of analyses to the yeast IGPS enzyme to demonstrate further capabilities of the *MDiGest* package, including the community network analysis. Cas9 allows demonstration of the use of the *MDiGest* package as applied to a complex system involving large displacements of multiple domains.

### A. Correlation networks in MptpA

MD simulations are particularly valuable for studies of allostery since they provide the history of atomic motions in terms of a time series of molecular configurations. Irrespective of the type of input coordinates or features that characterize each time step, only a subset of them are typically most involved in a specific biomolecular process. A general approach to identifying the coordinates most relevant to a process is to consider their mutual relations as quantified by a measure of correlation. Differences in the correlation profiles between two different states (i.e., wild-type and mutant) reveal the effect of that specific perturbation (mutation) on the overall dynamics of the system. *MDiGest* allows for efficient computation and comparison of different correlation metrics through correlation heatmaps and the visualization of correlation networks. Different measures of correlation, however, may provide different yet complementary types of information. To give a sense of the extent of such variations, we compare different metrics for three independent 200 ns replicas of two states of MptpA, including wild-type (WT) and the Q75L mutant. Through this analysis, we rationalize the effect of the mutation and, in turn, illustrate the reasons for the observed improved catalytic activity of Q75L.

**FIG. 3.**
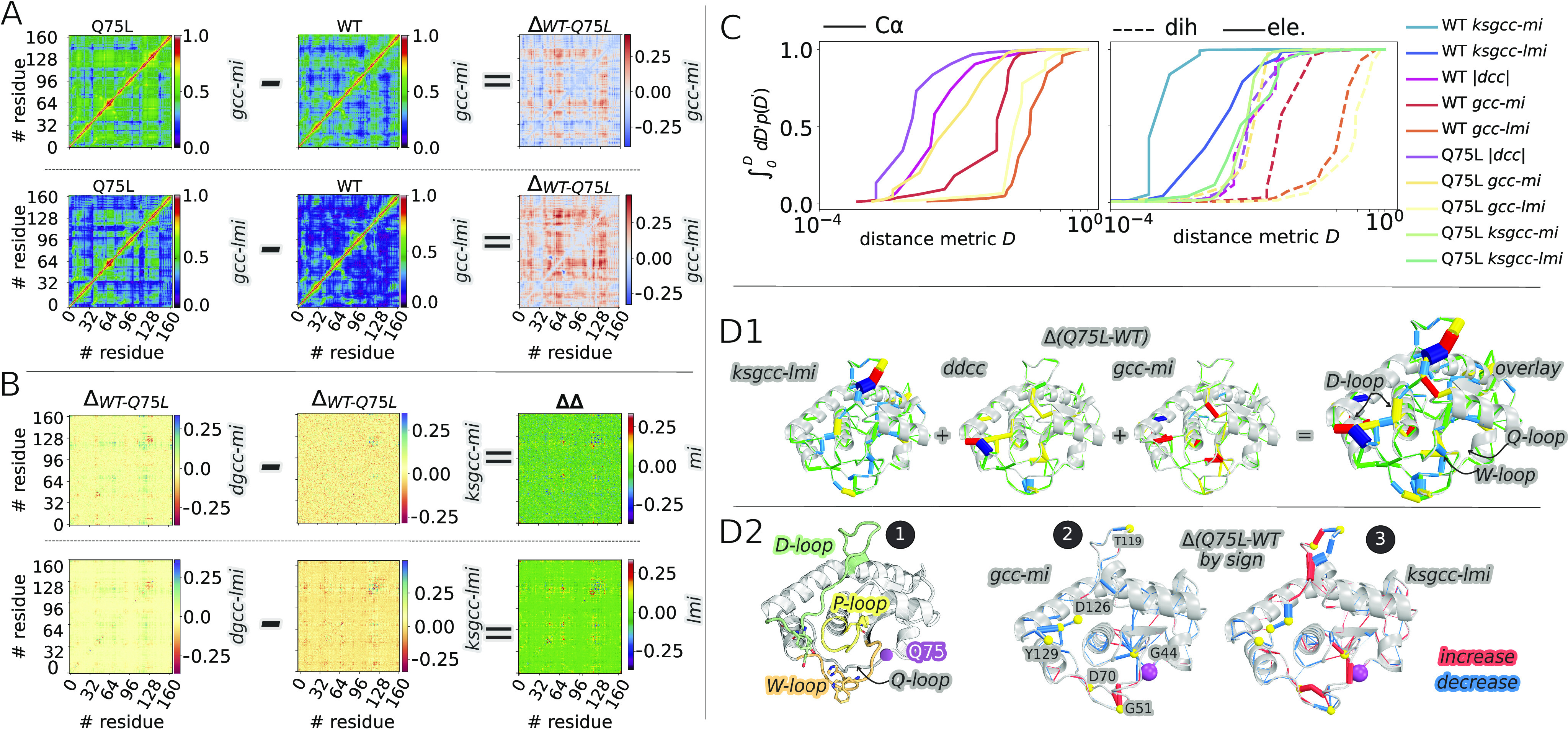
Correlation networks from different metrics. (a) Heatmaps of *lmi*- and *mi-gcc* correlations computed from Cα-displacements from simulated trajectories of WT and Q75L and their differences. (b) Heatmaps of difference ∆_WT-Q75L_ correlations computed from dihedral distributions and electrostatic energies using *mi*- and *lmi*-based correlation metrics. (c) Cumulative distributions of the log distributions of various similarity measures D = −log(C). (d1) Several difference networks from Q75L minus WT linearized mutual information-based correlation of electrostatic energies (*ksgcc-lmi*), dynamics cross-correlation of dihedral fluctuation (*ddcc*), and mutual information-based generalized correlation coefficients from Cα-displacements (*gcc-mi*). Each network is filtered according to the average distance matrix computed from the Q75L and WT trajectories. Only edges corresponding to Cα-pairs lying at a distance lower than or equal to 5 Å are retained. Moreover, the edges are distributed in five equally spaced bins ranging from 0 to 1 and colored accordingly. A consensus network obtained by overlaying the *gcc-mi*, *ddcc*, and *ksgcc-lmi* networks is also shown. (d2) 1: MptpA enzyme with the three catalytic loops. Color key: P-loop (yellow) with the catalytic C11 shown in sticks; D-loop (green) featuring Y128 and Y129 in sticks; and W-loop with W48 in sticks (orange). The Q75 residue in the Q-loop is shown in a magenta sphere. 2: Difference networks (Q75L minus WT): *gcc-mi* and *ksgcc-lmi* colored by sign. Edges that experience an increase in correlation upon mutation are shown in red, while edges experiencing a decrease in correlation are shown in blue.

Figure 3(a) shows the averaged *gcc* matrices computed from Cα displacement sampled from MD trajectories of WT and Q75L MptpA. In the first row, the pairwise couplings (*gcc-mi*) are estimated using a non-parametric *k*-nearest neighbor density estimator (with *k* = 5), which accounts for non-linear correlations. In the second row, the correlation (*gcc-lmi*) is computed assuming the special case of a Gaussian joint probability distribution that only captures the linear regime. The latter approach drastically reduces the computational effort, however, at the cost of neglecting non-linear terms. The difference correlation matrix (Q75L-*minus*-WT) appears to overestimate the overall gain in correlation due to mutation. Panel (b) compares different correlations computed from dihedral fluctuations (*dgcc-mi* and *dgcc-lmi*). Notably, using dihedrals in place of Cα atoms reduces the differences between the non-linear and linear metrics.

The difference Q75L-*minus*-WT dihedral correlations are compared to those derived from electrostatic energies (referred to as *ksgcc* and obtained using the Kabsch–Sander formalism described in Sec. II). The *ks*-picture compares very well with the correlations obtained from dihedral distributions. The difference between *dgcc* and *ksgcc* shows that the delta-correlation patterns associated with the mutation are conserved regardless of the inclusion of non-linearities. A quantitative comparison of the correlation distributions obtained from different metrics is shown in Fig. 3(c). Here, we express the correlations in terms of “distances” by transforming the correlations (*C*) into *D* = −log(*C*).

Smaller distances correspond to larger correlations. The curves in panel (c) represent the cumulative probability of each distance distribution. The left panel shows the distance distribution computed from Cα displacements using *gcc*-*mi*, *gcc-lmi*, and *dcc* (time-averaged Pearson correlation introduced in Sec. II) for WT and Q75L. The two linear metrics (*dcc* and *gcc-lmi*) behave rather differently, with the former having more than 90% of the values lying below 0.01. *gcc-lmi* is also steeply distributed, but toward larger values. *gcc*-*mi* accounts for small and high correlations, so it should be the preferred choice among the three-correlation metrics. The right panel shows a similar analysis, where the correlation metrics are computed from dihedral displacements (shown as dashed lines). In addition, these distributions are compared to those obtained by applying the *mi* and *lmi* metrics to the time-evolution of electrostatic energies (denoted as *ksgcc-mi* and *ksgcc-lmi*). Again, for dihedrals, *mi*-based metrics are more evenly distributed across the [0,1] interval and are comparable to the *ksgcc-lmi* distributions. To create a consensus picture, we overlay the networks obtained with different metrics that have the largest overlap [Fig. 3(d)]. The different edge colors reflect the binning of each distribution, making it easier to single out the edges that are more significantly perturbed upon mutation.

Remarkably, the overlay between the different metrics [Fig. 3(d1)] captures all the relevant features at once, where the W-loop, Q-loop, and D-loop exhibit the largest change in correlation. Upon mutation of Q75 to L, the missing Q sidechain breaks a hydrogen bond between T45 and Q75, which is accompanied by a decrease in correlation in the neighboring region [blue lines around Q75 in Fig. 3(d2.2)]. In the electrostatic network, this change increases the electrostatic coupling (red lines) due to the formation of intermittent hydrogen bonds between the backbone of G44 and Q75.^30^ Overall, the Q-loop and W-loop remain coupled, but with a decrease in cross-loop electrostatic interactions in favor of increased interactions within the Q-loop [Fig. 3(d2.3)]. These motions promote a conformational change that brings the D-loop in close proximity to the W-loop, stabilizing the closed conformation essential for catalysis.^30^

### B. Community detection in yeast IGPS

Another common way of analyzing dynamic networks is by looking at the communities formed by the network nodes. In molecular systems, such analysis can identify protein domains that are functionally connected and elucidate key amino acid residues that establish the interactions between communities. Qualitatively, a community is defined as a subset of nodes that are more interconnected among themselves than with other nodes in the network. Therefore, pairs of nodes are more likely to be correlated if they are both members of the same community. Identifying such communities provides a detailed characterization of complex patterns of relationships between different regions of the protein.

The community detection module in *MDiGest* applies the Louvain heuristic,^65^ which is an unsupervised algorithm based on modularity optimization and community aggregation that allows for the efficient detection of communities. Despite efficiently solving the community problem, even for large systems, the Louvain algorithm has a major drawback: it often yields arbitrarily weakly connected communities. In some cases, communities may even be disconnected, especially when running the algorithm iteratively. To solve this issue, *MDiGest* includes an additional aggregation step where the nodes assigned to partitions smaller than a given threshold are iteratively reassigned to the community that yields the highest modularity.

**FIG. 4.**
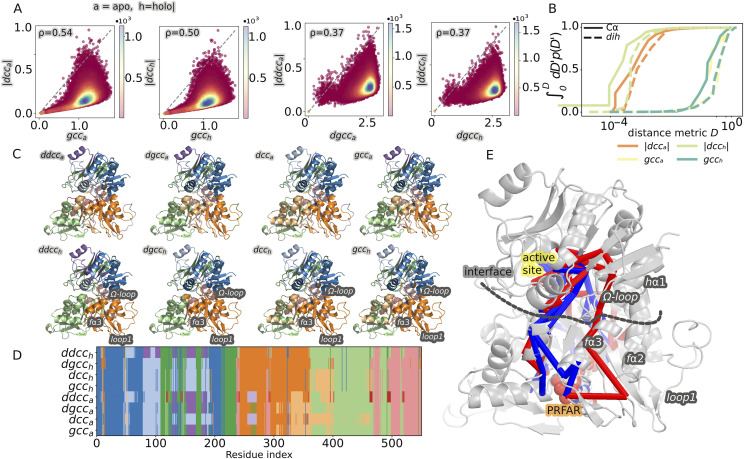
Community network analysis of yeast IGPS. (a) Linearized mutual information computed from the Cα and dihedral dynamics (*gcc*, *dgcc*) compares well to the absolute dynamical cross-correlation coefficient, as obtained from both features (|*dcc*|, |*ddcc*|) for apo (*a*) and holo (*h*) states. All distributions are on a log scale. ρ values denote the Spearman correlation coefficient between each pair of distributions. (b) Cumulative probability distribution for different measures of correlation. (c) Communities obtained according to the different metrics and for the apo and holo states projected onto the secondary structure of IGPS. (d) Representation of the communities as a function of the residue index for both apo and holo states obtained with different metrics of correlation. (e) Representation of the shorter pathways interconnecting the residues that form stable interactions extending from PRFAR to the active site. Pathways obtained from the apo simulations are shown in blue, as opposed to those obtained from the holo simulations, which are shown in red.

Figure 4 shows the results of community detection for the yeast IGPS enzyme as applied to studying how the effector (PRFAR) perturbs the correlations in the system. To do so, we analyze MD simulations of six independent trajectories 200 ns long of apo and PRFAR-bound IGPS. We computed various correlation metrics using *MDiGest*, including *gcc* from Cα and dihedrals and *dcc* (respectively denoted as *gcca/dgcca* for apo and *gcch/dgcch* for holo states). Figure 4(a) shows the correlation between gcc and |*dcc*|, as well as *dgcc*, and |*dcc*| for the apo and holo states computed from the average distributions over six independent replicas. The resulting contour plot reveals a clear relationship between the two correlation measures. Quite similar results are found for both the apo and holo Cα-based distributions (Spearman ρ = 0.55), suggesting that the two metrics are rather interchangeable. The correlation is slightly lower (Spearman ρ = 0.37) when computed from dihedral fluctuations, implying that the two metrics characterize some of the correlations differently. Indeed, the values for *gcc* extend over the full range from 0 to 1, while the values for *dcc* are very small compared to those of *gcc*. This effect is shown in more detail in Fig. 4(b), where the cumulative probability distributions obtained from the different metrics are shown, confirming that over 90% of the values computed for dihedral-based *dcc* lie below 0.01. The *dcc* distributions, however, seem to discriminate better between the apo and holo states. This analysis clearly demonstrates that different metrics can provide different information. Therefore, it is important to verify the consistency of the communities as obtained from different measures of correlation.

Figure 4(c) shows the projection of the communities computed from different metrics onto the protein structure. Despite the differences discussed previously, the partitions are rather consistent regardless of the chosen metric. Remarkably, all metrics show that upon the addition of PRFAR, the *loop1*-*f*α2-*f*α3-*Ω*-*loop* forms a single community, which is consistent with the functional role of such residues detailed in previous experimental findings.^34,68^ The addition of PRFAR promotes changes in interactions that propagate from the effector site to the Ω-loop and affect the inter-domain interface, initiating the rearrangement of the active site that culminates with catalytic activation.^69^ The signal transmission is assisted by the motion of loop 1. PRFAR increases the internal correlations in the protein, resulting in reduced fragmentation of the partitions. The analysis of shortest pathways (based on the Floyd–Warshall^70^ algorithm applied to the *dgcc*_*a*_ and *dgcc*_*h*_ networks) confirms that upon the addition of PRFAR, the allosteric signal transmission from PRFAR to the active site involves *f*α3, *loop1*, and the *Ω*-*loop*.

### C. Community detection in Cas9

The analysis of the Clustered Regularly Interspaced Short Palindromic Repeats-associated 9 (Cas9) enzyme demonstrates the utility of different analyses provided by the *MDiGest* software package. Specifically, the analysis shows how to use *MDiGest* to elucidate the allosteric pathways in Cas9 and how they change upon DNA binding. The eigenvector centrality values obtained from the *gcc-lmi* correlation coefficients of KS energies describe the level of participation of each node during the propagation of electrostatic interactions along the network. This protocol allows for the identification of the nodes that are crucial for transferring electrostatic interactions through the network. The comparison of the allosteric network of Cas9 with and without bound DNA provides insight into the allosteric mechanism of Cas9 with DNA. HNH is identified as a hub for signal transmission, with strong edges that connect HNH radially to the neighboring domains (RuvC), PI, and REC.

**FIG. 5.**
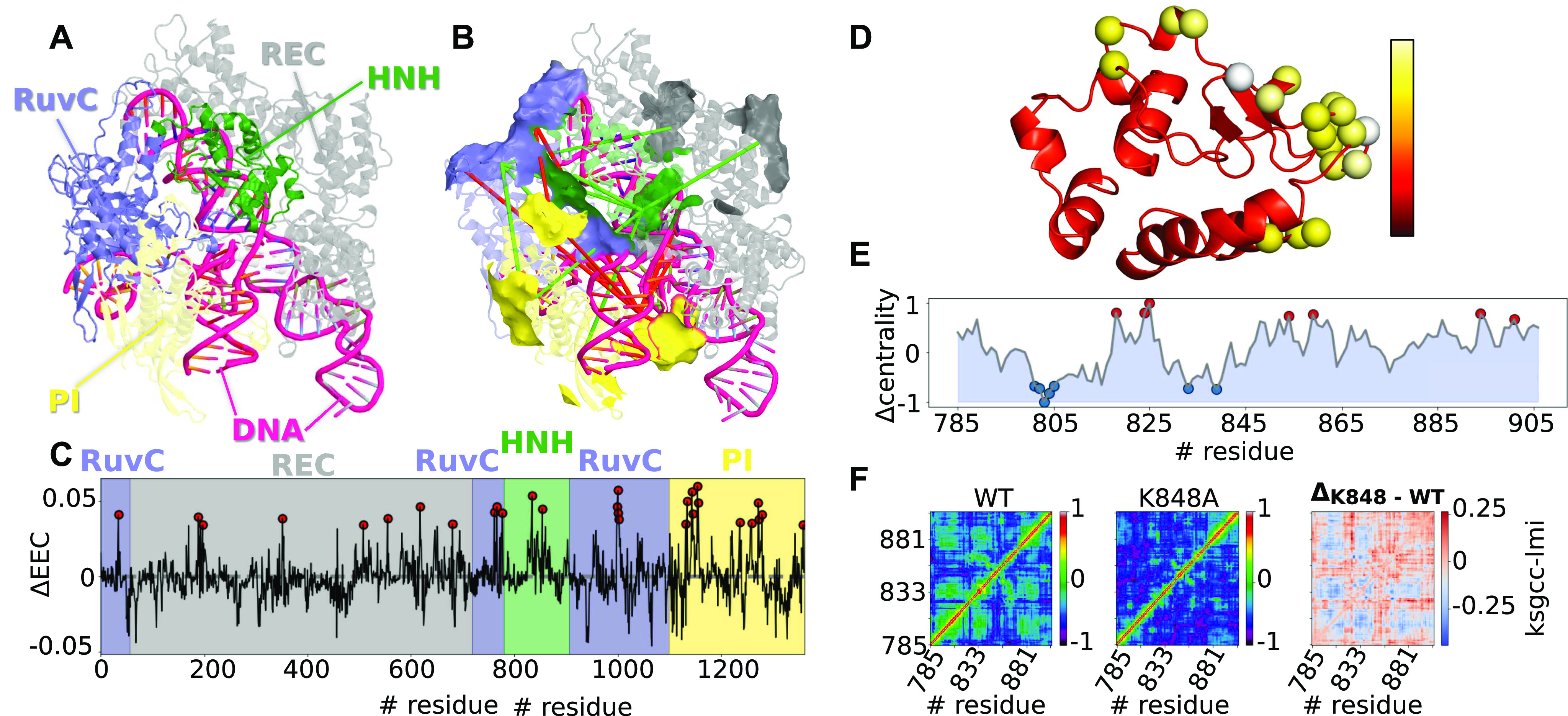
Network analysis of Cas9. (a) Cas9 protein with colored subunits: REC = gray, RuvC = blue, HNH = green, and PI = yellow. (b) Electrostatic network of Cas9, where nodes correspond to secondary structure clusters and edges are defined by the change in the correlation of KS electrostatic energies induced by DNA binding (green edges = increase, red edges = decrease). (c) Changes in electrostatic eigenvector centrality values (∆EEC) of Cas9 induced by DNA binding. Residues with ∆EEC greater than two standard deviations above the mean are shown as circles, indicating the residues that become significantly more central to the propagation of electrostatic information upon DNA binding. (d) Cas9 HNH K848A mutant colored by RMSF. Residues with RMSF larger than two standard deviations above the mean are shown as spheres. (e) Change in eigenvector centrality values computed from correlations of α-carbon displacements between wild-type (wt) HNH and HNH mutant K848. (f) Correlation plots for wt HNH, K848A, and their differences.

The difference (DNA-bound *minus* apo) in electrostatic eigenvector centrality [Fig. 5(c)] identifies residues whose centrality in the electrostatic network is significantly enhanced and thus becomes more important in the allosteric pathway upon DNA binding. Additionally, we can create a network representation of Cas9 where nodes correspond to secondary structure clusters (i.e., adjacent residues with the same secondary structure during most of the MD simulation), and edges between nodes are defined by pairwise *gcc*s computed from KS energies throughout the trajectory. Since we are interested in describing the effects of DNA binding on the Cas9 allosteric mechanisms, we use the difference in correlation coefficients between DNA-bound and apo Cas9 as the edges, where the width of the edge is proportional to the magnitude of the change and the color (green = increase, red = decrease) denotes the directionality of the change [Fig. 5(b)].

Figure 5 shows that the HNH domain, for example, becomes more central to the electrostatic network upon DNA binding, as characterized by both the electrostatic eigenvector centrality and electrostatic network analyses. Zooming into the HNH domain, we compare wild-type HNH with a mutant that has had an allosteric hotspot (residue 848) mutated from lysine to alanine (K848A). The eigenvector centrality from correlations of Cα displacements of the two states (wild-type and mutant) reports on the change in allosteric communication upon the point mutation. These results identify the residues whose centrality to the network changes significantly [Fig. 5(e)], as well as the changes in pairwise correlations [Fig. 5(f)], consistent with previous studies.^6,71,72^

## V. PERFORMANCE

**FIG. 6.**
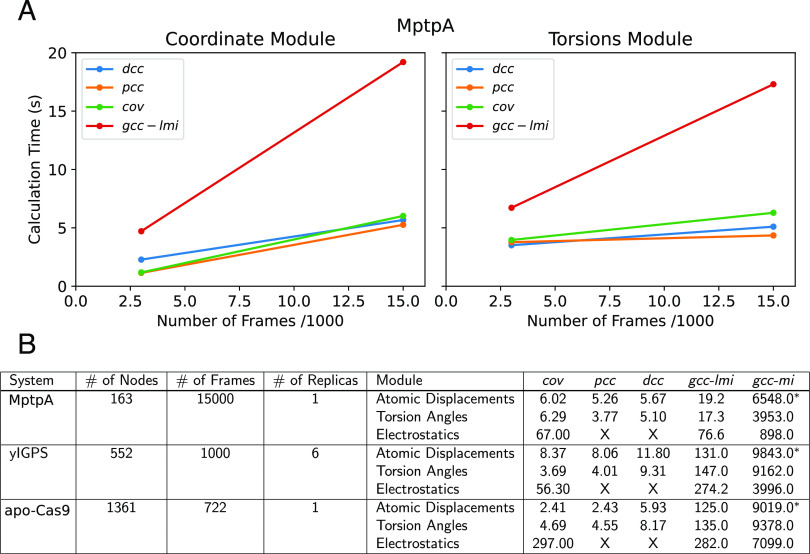
Benchmark of performances. (a) Benchmark calculations measured by calculation time (CPU clock time) in seconds, computed on GoogleColab using TPU runtime—Intel Xeon CPU @2.30 GHz, 32 GB RAM, and a cloud TPU with 180 teraflops of computational power. (a) Comparison of the calculation time as a function of the number of frames in the trajectory using the following metrics: the dynamical cross correlation (*dcc*), Pearson correlation (*pcc*), covariance (*cov*), and generalized correlation coefficient derived from linearized mutual information (*gcc-lmi*) computed from atomic coordinates (left) and dihedral angles (right) for the MptpA trajectory using a total of 3000 frames and 15 000 frames. (b) Table summarizing the approximate calculation times for different benchmark systems using the three main modules of MDiGest. The correlation metrics are the same as defined for Panel (a), with the addition of a generalized correlation coefficient derived from mutual information (*gcc-mi*). The asterisks are used to denote the use of two different parameters in the calculation for *gcc-mi*, where the asterisk in the Atomic Displacements rows reflects the use of *knn_5_1* [*implying* using *k* = 5, setting 1/k in Eq. (12) 0 and including in the nearest neighbors count the node for which the KDTee is computed], while those without the asterisk are computed using *knn_5_2* (computed excluding the node relative to which the neighbors search is performed and retaining the 1/k term).

Figure 6 reports the timings for different correlation calculations using the different modules available in *MDiGest*, providing a reference for how computation scales depending on the number of nodes/frames included. Generally, for an equal number of frames (*F*) and nodes (*N*) considered, the calculation of Pearson correlation coefficient (*pcc*) and dynamical cross-correlation (*dcc*) matrices is comparable to that of covariances (*cov*) for dihedral and atomic-displacements and slightly faster than computing linearized-generalized correlation coefficients (*gcc-lmi*). For example, the time taken for calculating *cov*, *pcc*, *dcc*, and *gcc-lmi* for atomic-displacement networks built from 163 atoms (MptpA) and 15 000 trajectory frames is 6.02, 5.26, 5.67, and 19.2 s, respectively. The linear scaling of the time consumption associated with these networks evaluated for MptpA is shown in Fig. 6(a).

The apparent increased time cost for computing *cov* and *gcc-lmi* networks based on the electrostatic coupling is due to the implicit computation of four *N* × *N* × *F* distance matrices (*dC*_*i*_*H*_*j*_, *dC*_*i*_*O*_*j*_, *dC*_*i*_*N*_*j*_, *dO*_*i*_*N*_*j*_) between the backbone atoms of each pair of residues *i*,*j* = 1, …, *N* that underpins the estimation of correlations. While the computation of these distance matrices is performed in parallel, electrostatic couplings networks are by far the most computationally demanding option available in *MDiGest*. However, once these distance matrices are computed, the calculation of mutual information-based correlation is faster on electrostatic couplings than on atomic displacements due to the three-fold reduction of dimensionality in the value arrays obtained when the per-frame electrostatic couplings are averaged over columns or rows to obtain the corresponding donor/acceptor time series.

The starred vs non-starred *gcc-mi* presented in Fig. 6(b) showcases the impact of two options, namely *option* = *1* vs *option* = *2*, along with *k* = *5* (number of neighbors) for the mutual information estimator in the *gcc-mi* calculations. The nearest-neighbor calculation involves considering *k*-neighbors for a given node with two options available: including or excluding the given node itself in the calculation, where the latter option includes an offset of 1/*k*. The scaling effect is observable in MptpA due to the considerable number of frames in a single replica. However, in cases where fewer frames are used, such as yIGPS and Cas9-apo, the difference between *options 1* and *2* is less noticeable.

*MDiGest* has a non-parallel architecture if we exclude the in-parallel computation of the distances in the KS electrostatic terms, and few instances include parallel array computation. Therefore, running *MDiGest* on Google Colab graphics processing unit (GPU) platforms will only positively affect those functions that make explicit use of JIT compilers. Future improvements will include explicit parallelization of the computation of the mutual-information-based correlation and computations over multiple replicas.

## VI. MDiGest IN COMPARISON TO OTHER EXISTING TOOLS

Despite the widespread use of protein correlation networks (PCNs), there is a lack of *standardized use* of such methodologies in terms of the selected correlation metrics and handling of MD trajectories, such that often one metric is selected over another somewhat arbitrarily. The resulting interpretations can thus become dependent on the choice of metrics. *MDiGest* allows for comparisons of analyses based on different metrics to obtain a consensus picture with respect to the identification of residues central to allosteric mechanisms.

Although many tools have been published in recent years to analyze correlations from MD simulations, the lack of standardization can hinder reproducibility. In addition, available tools are limited with regard to their input formats and supported network models. For instance, packages such as *MDTraj*,^62^ *pytraj*,^15^ *MDAnalysis*,^31^ and *ProDy*^73^ allow for efficient parsing of MD trajectories and large-scale preliminary general analyses. However, those packages do not provide PCN analyses available in other Python packages such as *correlationplus*,^10^ *MD-TASK*,^11^ *MDEntropy*,^13^ *dynetan*,^18^ *and MoSAIC*.^74^

*Correlationplus*^10^ consistsv of a set of Python scripts to compute correlations based on *gcc-lmi*, *dcc*, and *pcc*. It is based on ProDy^73^ for processing MD trajectories, while *MDiGest* is built on *MDAnalysis*.^31^ *Correlationplus* does not compute (nonlinear) *mi*-based correlations while providing access only to the analysis of linear correlations and integrates MD-correlation with normal model-based analysis.^75^ *MD-TASK*^11^ (and the corresponding web server, *MDM-TASK-web*) is among the most comprehensive software suites for coarse-grained analysis of allosteric proteins. It provides a variety of non-conventional approaches, such as dynamic residue network analysis, perturbation-response scanning, dynamic cross-correlation, essential dynamics, and normal mode analysis, thereby integrating common approaches to static and all-atom MD-simulated proteins. However, it lacks the incorporation of MI-based correlations. *Dynetan*,^18^ on the other hand, provides an efficient implementation for generalized correlation based on mutual information and associated network analyses. However, it only allows network construction from atomic displacements, which makes it rather limited in terms of feature space. *MDEntropy*,^13^ as the name suggests, is a collection of modules for performing information-theory-related analyses on MD trajectories. However, it is unclear whether it is actively maintained. Another package that allows a systematic comparison of correlated motions from MD simulations is *MoSAIC*,^74^ which uses block-diagonalization of the correlation matrices to extract relevant collective motions underlying functional dynamics from uncorrelated motions. This strategy avoids possible bias due to presumed functional observables and conformational states or variational principles that maximize variance or timescales.

With simulations growing longer and larger, capturing correlated behavior in the presence of large motions is becoming increasingly important for properly understanding protein dynamics. Those applications require the use of internal coordinates rather than atomic displacements, where the latter depend on the reference frame used for trajectory alignment. Different definitions of coordinate-independent metrics have been proposed, including correlation of all rotameric and dynamical states (*CARDS*),^14^ protein contact networks (*pmdlearn*),^17,26^ and interaction networks (*gRINN*).^9^ These are all complementary approaches that can be used in conjunction with PCNs, all with their own caveats: the motion of adjacent backbone dihedral angles is necessarily correlated in a protein with relatively rigid secondary structures (the “loop closure problem”).^27^ Protein interaction and contact networks, on the other hand, require distance cutoffs to define whether any two residues are *interacting* according to threshold hyperparameters that have to be carefully tuned for optimal results.

To deal with these limitations, we proposed a different approach based on the construction of protein-correlated electrostatic couplings, which are available in *MDiGest* in the KS-correlation module. The interaction energy terms are built through the KS^53^ formalism, which accounts for donor-acceptor cross-residue amide-to-carbonyl backbone Coulombic interaction terms. In an earlier study,^30^ we showed that the distribution of correlated electrostatic couplings is highly correlated with that of predicted chemical shifts based on the conformational ensemble obtained via MD simulations. In fact, the average predicted shifts were shown to be highly correlated with experimental chemical shifts. Therefore, it was shown that the correlated electrostatic couplings are predictive of which amino acids may be most sensitive to changes in a dynamical ensemble and provide improved correspondence to NMR experiments. A related software, *PyInteraph2*,^16^ offers another tool designed to analyze MD and structural ensembles with an emphasis on pairwise interactions between residues, including hydrogen bonds, salt bridges, and hydrophobic interactions, but differs from *MDiGest* in that it does not calculate correlations. Another recent tool is *AlloViz*,^12^ a wrapper that combines some newly written modules aimed at providing a general framework for calculating residue interaction networks. The benefit of this tool is that it incorporates a handful of options for constructing the protein network, although its capabilities for network analysis are limited. Aside from Python-based tools, the Bio3D Package provides a wide range of utilities to analyze, process, organize, and explore biomolecular structure, sequence, and dynamics data based on R. Bio3D comes with various correlation analysis and accessory functions allowing for statistical analysis and visualization of biological sequence and structural data.^76^

The description above is inclusive merely of the subset of allostery-related methodologies that have been included in recently developed distributed packages while excluding complementary approaches. Among these, Lake *et al.*^77^ proposed the use of a protein graph Hessian constructed from the residue position covariance matrix and average structure upon application of a force constant that is optimized to reproduce pairwise particle variances produced in the simulated MD ensemble. In contrast to correlation metrics such as MI-based generalized correlations or Pearson correlation, Hessian elements are only finite for short-ranged physical interactions, thereby providing direct access to identify those short-ranged interactions that lead to long-ranged correlations.

The list above is a conspicuous, although non-exhaustive, collection of recent developments related to the study of allosteric interactions in protein networks, the extent of which is a testimony to the central role played by short-range interactions in the functional regulation of proteins. This motivates the need for additional multivariate descriptors that yield an unequivocal measure of allosteric interactions.

Moreover, the field of allostery is still lacking consensus with regard to a general procedure to design and implement network models. Efforts to develop platforms and strong foundations to study protein networks of highly dynamical biomolecules are still needed. This is especially relevant because of the potential of protein network approaches to complement experimental studies of a wide range of systems. For example, PCNs and interaction networks may help design catalytically enhanced enzymes, identify selective druggable allosteric hotspots for selective inhibitors, or even assess the impact of disease-related variants.

In an effort to contribute to the development of harmonized and reproducible protocols, *MDiGest* offers a self-contained and easy-to-use package based on a modular structure, allowing straightforward comparison of PCNs. For the same topology and MD trajectory, one can choose to construct correlation networks based on atomic or dihedral fluctuations or through the correlation of electrostatic interaction energies. Moreover, for atomic fluctuation-based correlations, the PCN can be constructed based on the movement of any combination of atomic selections or the center of mass of each residue. Implemented correlation metrics for atomic displacements, torsional fluctuations, and electrostatic-based networks are covariances, Pearson’s correlation coefficient, dynamic cross-correlation, Mutual Information (MI), or Linear MI (LMI). Conveniently, one can save the output of the correlation analysis for later use.

*MDiGest* provides various methods that can be analyzed and compared all at once through inspection of 2D correlation plots and their corresponding eigendecompositions, including direct visualization of the PCNs at custom correlation thresholds. Investigating the effect of a given perturbation (mutation or binding of the effector) on the allosteric dynamics is straightforward through the construction of difference networks. Finally, the PCNs can be analyzed through various algorithms for community network analysis, including the Girvan–Newman algorithm, the Louvain heuristic algorithm, and a newly proposed approach based on grouping highly connected secondary structure blocks. Furthermore, *MDiGest* provides a visualization module to plot and interactively analyze PCNs in PyMOL.^20^

We foresee that the modular structure of *MDiGest* as a Python package made freely available through GitHub will contribute to promoting a community-driven effort focused on boosting reproducibility and establishing standardized protocols in the PCN field. As developers, we are committed to continually introducing new functionalities, training new contributors, providing assistance to users, and maintaining the *MDiGest* package.

## VII. SIMULATION DETAILS

Details related to the MD simulations of MptpA and IGPS are reported in Refs. 30 and 69, respectively. Detailed descriptions relative to the Cas9 HNH mutant and full Cas9 MD trajectories are reported in Ref. 6. A brief description of the simulation protocols used for each system is reported below.

**MptpA**. The structural models for the wtMptpA and Q75L simulations were established on the basis of the 1U2P 1.9 Å resolution x-ray structure.^78^ MD simulations were performed using the AMBER-ff14SB^79^ force field for the protein, as included in the Amber20 software package.^80^ We executed eight distinct MD simulations, four for each system, for a total simulation span of 0.8 *µ*s, as previously described in Ref. 30. Refinements such as adjusting protonation states and adding explicit TIP3 water solvent molecules (up to density values ≥0.9 mol Å^−3^) were performed via AmberTools (2020).^80^ MD simulations were prepared as follows: first, we minimize the solvent by restraining all atoms but water and ions at the crystal structure positions. Optimized solvated structures were then gradually heated to 303 K, performing MD simulations (of 1 ns at least) in the canonical NVT ensemble using Langevin dynamics. Unconstrained MD simulations were carried out for 45 ns, resulting in a total pre-equilibration simulation time of about 50 ns. The pre-equilibrated systems were then simulated in the NPT ensemble at 300 K and 1 atm using Langevin dynamics for 200 ns. All simulations were performed using periodic boundary conditions with a switching distance cutoff of 10 Å.

**yIGPS**. The structural models for the apo and PRFAR-bound yeast IGPS complexes were established on the basis of the bienzyme complex from Sc-IGPS at a resolution of 2.4 Å (PDB: 1OX6-B), 1OX4-B,^81^ and 1JVN-A,^82^ in an effort to reconstruct from crystal models flexible residues that were unresolved in 1OX6-B. To complete the structure, six different structural models were generated through the use of different online tools, one via Modeller^83^ and another via Swiss-Model,^84^ with four suitable homology models found on ModBase.^85^ PRFAR was then bound to each model by aligning each structure to the effector-bound crystal structure of yeast IGPS (PDB: 1OX5).^81^ The 12 created structures (six in the apo state, six bound to the effector) had an RMSD of less than 5 Å. To compare the dynamics of IGPS enzymes from Tm- and Sc-IGPS, the simulation conditions were kept identical to those used for bacterial IGPS in Ref. 86, using a temperature of 298 K for the simulations. This choice was motivated by recent studies demonstrating that PRFAR has weaker allosteric activation at growth temperature than it does at room temperature.^87^ The simulations were conducted using the AMBER-ff99SB^88^ force field for the protein and the Generalized Amber Force Field^89^ for the PRFAR ligand, implemented in the Amber20 software package.^80^ 12 independent MD simulations were run for a total simulation time of 1.2 ms, each for a different complex (apo and PRFAR bound). Furthermore, details of the pre-equilibration procedure and MD production runs are provided in the original Ref. 69.

**Cas9.** MD simulations of Cas9 in apo form (apo Cas9) and in combination with DNA (Cas9:RNA:DNA) were carried out using the crystallographic coordinates of Streptococcus pyogenes apo Cas9 (4CMQ) and Cas9:RNA:DNA (4UN3), respectively, which were resolved at 3.09 and 2.58 Å. The simulations utilized the Amber ff12SB force field with Åqvist parameters for Mg ions, which favor an octahedral coordination of the Mg ion.^90^ The TIP3P model 33 was applied to parameterize the solvent. The Cas9 MD simulations have been performed with NAMD 2.10.^91^ under the isothermal–isobaric (NPT) ensemble and using a time step of 2 fs. The systems were simulated using a Langevin thermostat at 298 K and a barostat at 1 atm. Periodic boundary conditions were applied. The particle mesh Ewald (PME)^92^ method was used to evaluate long-range electrostatic interactions, and a cutoff of 12 Å was used to account for the van der Waals interactions. The complete description of the parameters and protocol applied in the simulations is detailed in the original publication by Palermo *et al.*^93^ The protein representations throughout the paper were generated using the PyMOL software.^20^

## Data Availability

All discussed analyses are implemented in the open-source software *MDiGest*, which is freely available at https://github.com/fmaschietto/mdigest/. The code necessary to reproduce the results presented in the paper is collected in the form of tutorial Jupyter notebooks available at https://github.com/fmaschietto/mdigest/notebooks/. Access to MptpA and IGPS simulated trajectories is provided within the *mdigest-tutorial-notebook.ipynb* notebook, in the same directory.
